# Abstract of the Proceedings of the Buffalo Medical Association

**Published:** 1862-03

**Authors:** J. F. Miner

**Affiliations:** Secretary


ART. II.—*Abstract of the Proceedings of the Buffalo Medical Association.*

TUESDAY EVENING, Feb'y 4, 1862.

Present—Dr. Samo, President *pro tem.* in the Chair, Drs. Rochester, Wyckoff, Kempson, Dayton, Cronyn, Miner, Congar, Washburn.

The Minutes of the last meeting were read, eliciting considerable discussion, in which most of the members present participated, and on motion of *Dr. Rochester* were approved as published.

Dr. Leon F. Harvey was unanimously voted a member on compliance with the by-laws.

*Dr. Rochester* reported a case of Puerperal Eclampsia, including in the history several items of interest bearing upon infecundity resulting from uterine disease, and subsequent pregnancy after an unusual interval. His remarks were as follows: "I was consulted in 1858 by a lady of middle age. She had been suffering for an indefinite period from leucorrhœa, irregular menstruation, and lumbar pain, and more recently from cystitis. She had borne two children, the youngest of whom was sixteen years old, since whose birth she had never been pregnant. Examination disclosed uterine enlargement, ulceration of the os and endo-metritis. General and topical treatment restored her to perfect health."

In October, 1861, I was again consulted. There was abdominal distension supposed to proceed from "change of life." On my second visit I detected the pulsation of the fœtal heart in the left hypogastrium. To my patient's surprise I pronounced her pregnant. She was unusually stout and plethoric, and from this time her gestation was carefully observed. The urine was repeatedly examined, but no trace of albumen could be detected. She was placed upon a somewhat restricted diet, ordered to take exercise in the open air, and to keep her bowels free with saline cathartics. These precautions were taken on account of her plethoric and florid aspect, although there was no œdema, and no headache. There was, however, frequent and distressing urticaria, a disorder I believe occasionally produced by uræmia. She was confined on the 15th of January, 1862, at 10½ P. M., after a short and easy labor of two hours' duration. Immediately after the birth of the child, a small but vigorous male, she took half an ounce of wine of ergot, given to secure uterine contraction and to prevent severe after pains. I left her at midnight, very comfortable. She was now 45 years old, and her third child was nearly 20 years the junior of her second. At 9½ A. M. on the 16th I found her very well. She had passed a quiet night, had slept some, and had *urinated* very freely; lochia abundant, but not excessive. She had experienced two or three strong after-pains, the last of which was followed by the discharge of a clot as large as a hen's egg. About half past 12 P. M. I received a very urgent message from her husband, and on my arrival at the house learned from Dr. G. F. Pratt, who had been called in, in the emergency, and who had kindly awaited my coming, that my patient, on waking from slumber, about 11½ A. M. had complained of slight headache, and immediately thereafter had a severe convulsion, from which she had hardly emerged when he reached her bed-side. Her mind was clear; she complained of dizziness; her face was flushed and her pulse was full and slow. Ice was, applied to the head, warmth to the extremities, absolute quiet and rest enjoined, and a small amount of chloroform was inhaled. At 12 M. she had another paroxysm, so severe and protracted, attended with extreme lividity and venous engorgement, that Dr. Pratt deemed it best to bleed her to the amount of twenty-four ounces. The venesection was followed by relief from the pain in the head, and the pulse became more soft and frequent. Cordially approving the treatment pursued by Dr. Pratt, I remained in the sick room, ready to act as circumstances might seem to require. The patient was quiet, free from pain, countenance natural, pulse 80, soft and compressible. On prosecuting my inquiries, I learned that for the six nights preceding her accouchment, she had risen from bed and held her feet for some minutes in *cold water* to relieve the intolerable burning and itching produced by the urticaria. That her bowels had been unusually confined for the past week, but that she had had, three copious dejections on the 15th, from a dose of castor oil, She had all the while urinated freely. At 1½ P. M., an hour and a half after the second convulsion, a third took place. It was not very severe, and was evidently lessened by chloroform which I obliged her to inhale. She emerged from it in a wild and restless condition, vomited freely, and continued for some time to strain and retch. To quiet her stomach, to secure catharsis and to obtain quiet, I gave at once, in combination, calomel grs. xv, sulphate of morphine gr. i. I also kept her steadily and continually under the gentle influence of chloroform for four hours, using two ounces of the article prepared by Duncan & Flockharty. Whenever there was the slightest manifestation of restlessness the anæsthetic was applied. At 6 P. M. I discontinued its administration, gave half a grain of morphine and left her under the supervision of my intelligent pupil, Mr. H. P. Babcock. At 8 P. M. I

found her sleeping quietly, and left her at 10 P. M. very comfortable, she having taken a little tea and expressed herself free from headache and all uncomfortable sensations. Some urine, the first she had voided since the convulsions, was now obtained; this was examined by my friend Prof. Hadley, who detected in it "a trace of albumen."

Jan. 17th, 9 A. M.—No unfavorable indications; the bowels have moved more freely; urine is abundant, and the lochial discharge has returned, after a suppression of sixteen hours. From this time convalescence was rapid; if the entire absence of the lacteal secretion, which never occurred, does not make the convalescence slightly incomplete."

*Dr. Rochester*, in remarking upon the treatment, said that the blood letting had relieved the headache, and although it had not prevented the return, had probably retarded and mitigated the severity of the eclampsia, and had perhaps facilitated the action of the other agents employed; that the calomel had arrested the vomiting and straining and had ultimately produced derivative catharsis, while the morphine and chloroform had maintained a continuous sedative effect, to which the recovery of the patient was probably due; and that to whatever the termination is ascribed, he was glad to place on record, one more escape from puerperal eclampsia.

*Dr. Kempson*, exhibited some vaccine lymph he had recently received from London, England. This lymph is placed upon points of bone or ivory; he regarded it as much more certain in its effects than the crust. Dr. K., offered to obtain it for any medical man who would call upon him; also offered the points on exhibition to any one who desired to make trial of them.

*Dr. Rochester* remarked that he had often called the attention of the members of the Association, to the superior qualities of vaccine lymph when compared with the virus contained in the crust; that for several years he had stood almost alone here advocating and using only lymph.

*Dr. Dayton* had noticed in the transactions of the Society, a few months since, that Dr. Kempson reported "that closure of the os-uteri was intentionally produced by some physicians of Canada, who have recourse to various methods to produce inflammation and complete closure of the orifice, and that such cases constitute the bulk of the business of some Canadian practitioners;" Dr. Dayton had referred to it for the purpose of inquiring something more of the particulars of such practice; would like to understand by what means such results could be obtained? and also something more of the physicians "high in reputation with the best classes in Canada"

who are yet practicing in this manner " under the guise of healing ulcerations of the os-uteri."

*Dr. Cronyn* was surprised at the report of Dr. Kempson: hoped it could not be truthfully reported of any truly medical man in Canada.

*Dr. Rochester* had noticed the report and thought it remarkable; knew of no means by which such a result could be obtained; spoke of actual cautery as incapable of producing such result, and knew of no means by which it could be accomplished.

*Dr. Kempson* replied that it was only reported, by some gentlemen in a railroad car, and he had too much delicacy to report such a thing; it is a branch of business he does not understand: has not consulted the practitioners about it; is not acquainted with the individuals and cannot tell anything about it.

Adjourned to Tuesday evening, March 4, 1862.

J. F. MINER, Secretary.

